# Unlocking Coma Assessments: Exploring Healthcare Professionals' Knowledge and Perception of the Full Outline of Unresponsiveness (FOUR) Score in Saudi Arabia

**DOI:** 10.7759/cureus.78145

**Published:** 2025-01-28

**Authors:** Ahmed Lary, Reema Abualnaja, Osama Khojah, Manar Betar, Abdulrazak M Sakhakhni, Badriah Alsabbagh, Danya A Aljafari, Salem K Baeshen, Abdulelah Fattani, Hosam Aljehani, Yasmeen S Alsulaiman, Thamer Alaifan, Maan Jamjoom

**Affiliations:** 1 Neurosciences, Ministry of the National Guard-Health Affairs, Jeddah, SAU; 2 Medicine, King Saud bin Abdulaziz University for Health Sciences, Jeddah, SAU; 3 Research and Development, King Abdullah International Medical Research Center, Jeddah, SAU; 4 College of Medicine, King Saud Bin Abdulaziz University for Health Sciences, Jeddah, SAU; 5 Department of Neurosciences, Ministry of the National Guard-Health Affairs, Jeddah, SAU; 6 Medicine, King Saud Bin Abdulaziz University for Health Sciences, Jeddah, SAU; 7 Intensive Care Unit, Ministry of the National Guard-Health Affairs, Jeddah, SAU; 8 Neurosurgery, King Saud Medical City, Riyadh, SAU; 9 Neuroscience, King Faisal Specialist Hospital and Research Center, Jeddah, SAU; 10 Neurology, King Fahad General Hospital, Jeddah, SAU; 11 Emergency Medicine, Ministry of the National Guard-Health Affairs, Jeddah, SAU; 12 Neurosurgery, King Fahad Hospital of the University, Imam Abdulrahman Alfaisal University, Dammam, SAU; 13 Neurology and Neurosurgery, Montreal Neurological Institute and Hospital, McGill University, Montreal, CAN; 14 Department of Neurosurgery, Weill Cornell University, Houston Methodist, Houston, USA; 15 Medicine, Fakeeh College of Medical Sciences, Jeddah, SAU; 16 Emergency Medicine, King Abdullah International Medical Research Center, Jeddah, SAU; 17 Applied Medical Sciences, King Saud Bin Abdulaziz University for Health Sciences, Jeddah, SAU

**Keywords:** coma, four score, full outline of unresponsiveness score, glasgow coma scale, perception

## Abstract

Background

Coma scales play a critical role in assessing the consciousness level of comatose patients, guiding clinical decisions, and predicting patient outcomes. Although the Glasgow Coma Scale (GCS) has been the standard for decades, the Full Outline of UnResponsiveness (FOUR) score offers a more comprehensive assessment. In this study, the awareness, knowledge, and utilization of the FOUR scores among healthcare professionals in Saudi Arabia were explored.

Methods

This multisite, cross-sectional study was conducted between January and April 2023 and involved physicians specializing in emergency medicine, neurology, neurosurgery, or intensive care. Participants completed a self-administered questionnaire.

Results

Among 335 participating physicians, only 33% (111) reported having prior knowledge of the FOUR score; 54% (60) of physicians in this group rarely or never used the FOUR score, largely owing to the perception that the GCS suffices (45%, 61), and a lack of awareness among other healthcare professionals (43%, 58). A significant proportion of physicians unfamiliar with the FOUR score have expressed a willingness to adopt alternative scoring systems, and 67% (148) were open to using a system evaluating brainstem reflexes. For respiration and intubation, 65% (143) and 85% (187) of the physicians were open to alternative scoring systems, respectively. There was a significant difference in knowledge between specialties, level of training, and previous neurocritical training (p-values <0.001, 0.032, <0.001, respectively).

Conclusion

This study revealed a notable gap in knowledge and utilization of the FOUR score in Saudi Arabia, a willingness to explore alternative systems for assessing consciousness, and an interest in comparative studies of various coma scales. Efforts to improve education about the FOUR score among relevant healthcare professionals in Saudi Arabia, in addition to exploring alternative systems, is suggested.

## Introduction

Coma is defined as a state of deep unconsciousness where the individual exhibits no appropriate responses to stimuli such as pain, light, or sounds and remains unawakenable, persisting for an extended duration [1]. The Glasgow Coma Scale (GCS), established in 1974, is a widely used tool for quickly assessing the functional status of comatose patients [2]. It consists of three main components: eye responses, motor responses, and verbal responses [2]. However, the GCS may not assess the verbal component in certain situations where patients are intubated, aphasic, aphonic, or have vocal cord injuries [3]. Moreover, it may not accurately gauge coma severity in cases involving abnormal brainstem reflexes, altered breathing patterns, or ventilator dependency [3,4]. In contrast, the Full Outline of UnResponsiveness (FOUR) score, introduced in 2005, includes eye responses, motor responses, brainstem reflexes, and respiration, each scored from 0 to 4 [4]. A higher total FOUR score (up to 16) indicates a better level of consciousness [4]. Studies have demonstrated that the FOUR score addresses GCS limitations by encompassing these four aspects, enabling the detection of subtle signs of consciousness and distinguishing between conditions like locked-in syndrome and vegetative state [3]. The efficacy of the FOUR score has been validated in evaluating patients with neurological conditions such as strokes and brain injuries [5]. The gap in knowledge about the FOUR score in healthcare settings is a critical issue that needs to be addressed. There is a widening disparity between what healthcare institutions know and what frontline caregivers actually implement. This knowledge-practice gap results in inconsistencies in care, inefficiencies in workflows, and challenges in meeting the increasing demands of the healthcare system. To bridge this gap effectively, it is essential to empower frontline caregivers, including residents and consultants, with the necessary knowledge and tools and to implement more effective measures such as the FOUR score. This research aims to assess healthcare professionals' awareness and understanding of the FOUR score across various medical centers in Saudi Arabia.

## Materials and methods

**Figure 1:**
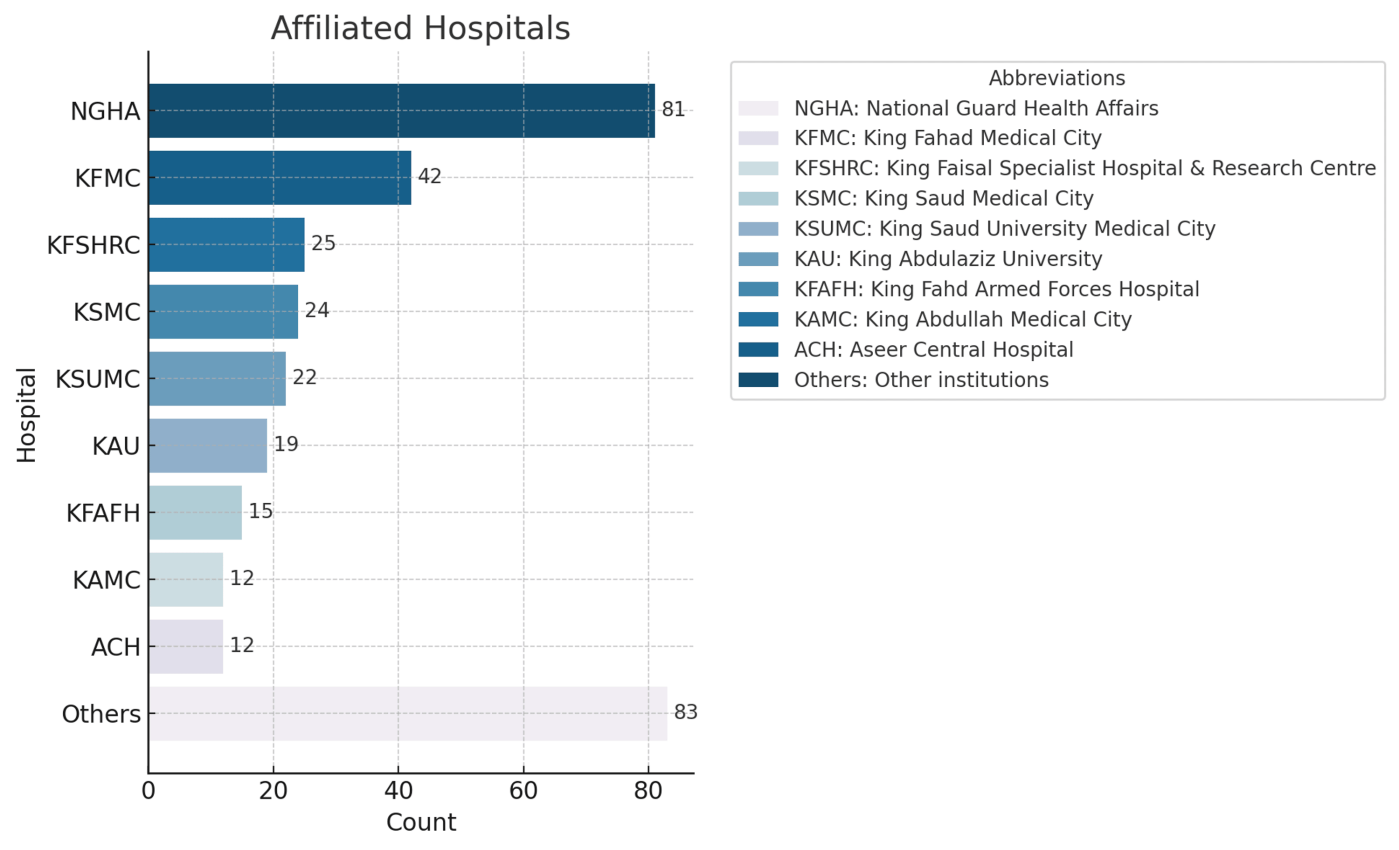
Overview of hospitals included in the study

In this multisite, cross-sectional study, the knowledge and perception of the FOUR score was assessed among physicians across various regions of Saudi Arabia. The study was conducted between January and April 2023 and involved 39 hospitals and institutions in Saudi Arabia, as shown in Figure 1. This study targeted physicians of all levels of training (residents, specialists, fellows, and consultants) specializing in neurology, neurosurgery, emergency medicine, or intensive care. Participants were selected from a comprehensive list of medical professionals registered with the Saudi Commission for Health Specialties (SCFHS). We included physicians licensed by SCFHS to practice one of the aforementioned specialties and excluded those who are no longer practicing or have changed their practice out of the specified specialties. These participants then received electronic invitations to complete a self-administered questionnaire. 

The survey comprised three sections, alongside queries on demographic data and training details. Initially, participants were asked if they had prior knowledge of the FOUR score. Based on their responses (yes or no), they were assigned into two groups: individuals already acquainted with FOUR score (group A) and those without previous knowledge of the score (group B).

For group A, the knowledge, clinical practice, and perception of the FOUR scores were explored. Their responses were graded on a five-point Likert scale, where '1' indicated minimal knowledge and '5' signified expert-level understanding of the FOUR score. Additionally, a frequency scale for using the FOUR score was obtained. The scale ranged from '1' (never used the FOUR score in general practice) to '5' (consistent application of the FOUR score in relevant cases).

On the other hand, group B shared their insights on the components of the FOUR score for assessing the level of consciousness, particularly on three key elements: brainstem reflexes, respiratory patterns, and evaluation of intubated patients.

The questionnaire was validated by a group of healthcare professionals before being distributed to the target population. The internal consistency and reliability of the questionnaire were assessed using Cronbach's alpha (α=0.87). A Cronbach's alpha coefficient greater than 0.7 was deemed acceptable for assessing internal consistency and reliability [6]. Data analysis was performed using the Stata v. 17.0 software (Stata Corporation LLC, College Station, Texas). The quantitative variables were presented as mean ± standard deviation. The association between two categorical variables was assessed using the chi-square test. A p-value of <0.05 was accepted for significance.

This study was approved by the Institutional Review Board of the King Abdullah International Medical Research Center (Review Board log No. NRJ22J/220/08), obtained in October 2022. Participants' completion and submission of the online survey were considered informed consent.

## Results

**Figure 2:**
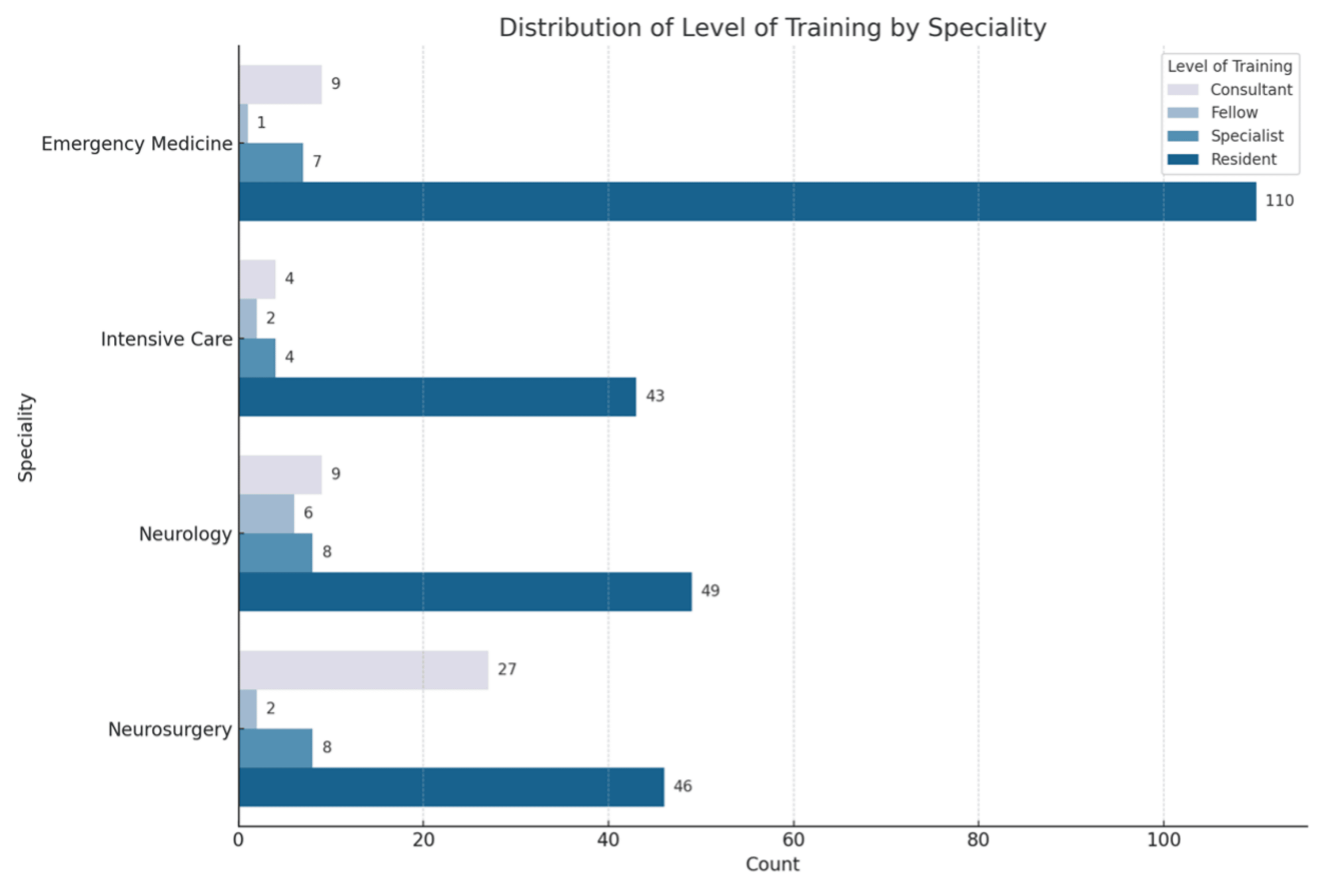
Distribution of level of specialties

A total of 335 physicians were included in this study, with males comprising almost two-thirds of the participants. Among the respondents, the largest group was residents (74%, 248), followed by consultants (15%, 49) and specialists (8%, 27) as illustrated in Figure 2.

The majority of respondents specialized in emergency medicine (38%, 127). There was no significant difference in knowledge between genders or consultants' years of experience (p-values: 0.983 and 0.387, respectively). However, there was a significant difference in knowledge between specialties, level of training, and previous neurocritical training (p-values: <0.001, 0.032, <0.001, respectively). The participants' characteristics and training details are presented in Table 1.

**Table 1:** Participants' characteristics and prior knowledge of the FOUR score FOUR - Full Outline of UnResponsiveness

Variable	With prior knowledge (group A) (n=111)	Without prior knowledge (group B) (n=224)	Total (n=335)
Gender			
Male	70 (63%)	141 (63%)	211 (63%)
Female	41 (37%)	83 (37%)	124 (37%)
Specialty			
Neurology	38 (34%)	34 (15%)	72 (21%)
Neurosurgery	27 (24%)	56 (25%)	83 (25%)
Intensive care	20 (18%)	33 (15%)	53 (16%)
Emergency medicine	26 (23%)	101 (45%)	127 (38%)
Level of training			
Consultant	16 (14%)	33 (15%)	49 (15%)
Fellow	7 (6%)	4 (2%)	11 (3%)
Specialist	4 (4%)	23 (10%)	27 (8%)
Senior resident	36 (32%)	73 (33%)	109 (33%)
Junior resident	48 (43%)	91 (41%)	139 (42%)
Consultant experience (years)	(n=16)	(n=33)	(n=49)
Less than a year	0 (0%)	3 (9%)	3 (6%)
1-5 years	5 (31%)	8 (24%)	13 (27%)
5-10 years	6 (38%)	6 (18%)	12 (25%)
10-15 years	2 (13%)	7 (21%)	9 (18%)
15-20 years	0 (0%)	4 (12%)	4 (8%)
20-25 years	2 (13%)	2 (6%)	4 (8%)
More than 25 years	1 (6%)	3 (9%)	4 (8%)
Neurocritical training			
Yes	65 (59%)	77 (34%)	193 (58%)
No	46 (41%)	147 (66%)	142 (42%)

Only 33% (111) of the respondents reported prior knowledge of the FOUR score (group A). Further analysis of group A respondents was conducted to assess their current understanding and implementation of the FOUR score in their clinical practices. The findings revealed that only 8% (9) of the participants were very familiar with the FOUR score, and only 3% (3) consistently used it in their practice. Notably, up to 54% (60) of healthcare professionals never utilized the FOUR score, and this was mostly attributed to the use of GCS instead (45%, 61) and lack of awareness of the FOUR score among other healthcare professionals (43%, 58). The perceived knowledge and clinical use of the FOUR score among group A are shown in Table 2. 

**Table 2:** Perceived knowledge and clinical use of FOUR score among group A a) respondents were allowed to choose more than one answer

Variable	Frequency (%)
On a scale from 1 to 5, how would you rate your knowledge regarding FOUR score?	
1	13 (12%)
2	23 (21%)
3	37 (33%)
4	29 (26%)
5	9 (8%)
On a scale from 1 to 5, how often do you use FOUR score in your clinical practice?	
1	60 (54%)
2	24 (22%)
3	19 (17%)
4	5 (4%)
5	3 (3%)
If you do not use FOUR score in your practice, why do you believe that is?^a^	
Using GCS is sufficient	61 (45%)
Lack of awareness among other healthcare professionals	58 (43%)
Perceived complexity and difficulty in use	7 (5%)
Inapplicability/Lacking importance	7 (5%)
Lack of training	2 (1%)
Concerns regarding its validation and the clarity of its interpretations	1 (1%)
Are you willing to start using it in your clinical practice?	
Yes	79 (71%)
No	32 (29%)

Nevertheless, most physicians in group A (88%, 98) supported conducting studies comparing the FOUR score to other coma scales. While a small percentage (12%, 13) expressed reservations, citing the existence of previous studies and time restrictions as reasons which they believed rendered further research unnecessary. 

Participants in group B were asked if they would use an alternative coma scale that is superior to GCS in certain factors (brainstem reflexes, respiration, and level of consciousness in intubated patients). Notably, 85% (190) of participants expressed willingness to adopt an alternative scoring system that evaluates the level of consciousness in intubated patients over GCS. Table 3 highlights the factors that might encourage participants to use the FOUR score over GCS.

**Table 3:** Preferences for using alternative scoring systems over the GCS for assessing consciousness among participants in group B GCS - Glasgow Coma Scale

Variable	Frequency (%)	
If there is a scoring system that evaluates brainstem reflexes to assess the level of consciousness, would you use it instead of GCS?		
Yes		151 (67%)
No		73 (33%)
If there is a scoring system that evaluates respiration to assess the level of consciousness, would you use it instead of GCS?		
Yes		146 (65%)
No		78 (35%)
If there is a scoring system that assesses the level of consciousness in intubated patients, would you use it instead of GCS?		
Yes		190 (85%)
No		34 (15%)

## Discussion

This cross-sectional study provides the first insight into the knowledge and perception of FOUR among relevant healthcare professionals in Saudi Arabia. The findings indicated that a substantial proportion of healthcare professionals are unaware of the importance of the FOUR score and its potential benefits. Knowledge of the FOUR score significantly differed based on specialty, level of training, and prior neurocritical training.

The present study is the first to explore the awareness, knowledge, and utilization of the FOUR score among healthcare professionals in Saudi Arabia. The findings of this study revealed a substantial knowledge gap, with only a third of respondents reporting prior familiarity of the FOUR score. This lack of awareness could be attributed to insufficient exposure to critical care training, especially considering that most respondents were residents (74%), with more than half of the residents being juniors (56%, 139). The foundational training for these residents, as outlined by the Saudi Commission for Health Specialties board programs, is structured to provide a broad base of medical knowledge. Yet, there is clear variation in the timing and extent of critical care and neurological education across different specialties. For instance, the neurology residency program is initiated following a year of general medical training, which offers limited critical care exposure [7]. Emergency medicine residents, meanwhile, only engage in a neuroscience rotation toward the end of their second year [8]. Similarly, neurosurgery residents are involved in neurological evaluations through preoperative assessments in their second year, while ICU residents are introduced to neurocritical training in their first year [9,10].

A study conducted by Yglesias et al. aimed to evaluate the assessment, awareness, and perception of nurses with varying levels of expertise on consciousness assessment using both the FOUR score and the GCS found that most participants were unfamiliar with the FOUR score and had not incorporated it into their practice [11]. Conversely, a similar study focused on evaluating the existing baseline knowledge of the FOUR score prior to any intervention [12]. It then aimed to assess the impact of structured educational programs designed to enhance this knowledge base [12]. Remarkably, prior to the educational interventions, an overwhelming majority of the participants (94%) self-reported only an average understanding of the FOUR score [12]. However, post-intervention, these same participants exhibited marked improvements in their knowledge and understanding, as evidenced by the study's outcomes [12]. This strongly supports the effectiveness of structured teaching in enhancing healthcare workers' proficiency with the FOUR score, pointing towards a practical approach for improving patient assessment and care. Over half the participants in group A (54%, 224) admitted having never applied the FOUR score in their clinical practice, with a preference for the GCS as the predominant reason. The advantage of simplistic systems, such as the GCS, is that they can be readily employed by various medical and paramedical professionals with fairly consistent reliability. This was also seen in the Yglesias et al. study, where nurses demonstrated a more profound familiarity and more consistent use of the GCS over the FOUR score [11]. They perceive the GCS to be extensively comprehensive, highly precise, and straightforward to administer, and therefore their preferred choice [11]. In comparison, while they acknowledge the FOUR score as detailed and accurate, they find it more complex to use and subsequently less favored in clinical settings [11].

Examining the brainstem reflexes could make the FOUR score perceived as a more difficult score or to require a more in-depth understanding of the nervous system in comparison to the GCS. It was also suggested that the interpretation of the breathing patterns could pose a challenge in mechanically ventilated patients [13]. However, a study assessing the perceptions of neuro/trauma ICU nurses highlighted a preference for the FOUR score over the GCS when assessing the neurological status of ICU patients. The nurses reported that the FOUR score was more accurate in identifying patients' neurological status, offered a more accurate assessment of intubated patients' true status, and yielded more objective results in terms of pupillary and respiratory evaluation compared to the GCS [14]. However, it is crucial to note that their study was limited by its small sample size (n=43), making it difficult to generalize their findings [14]. Our study explored the willingness of healthcare professionals in group B to adopt alternative scoring systems over the GCS for evaluating the level of consciousness, specifically in three distinct areas: brainstem reflexes, respiration, and assessment of intubated patients. Firstly, when considering the incorporation of brainstem reflexes into the assessment of consciousness, a significant majority (67%, 151) indicated a preference for such a scoring system. This suggests a recognition of the importance of brainstem reflexes in determining consciousness levels. Monitoring brainstem reflexes is crucial in traumatic and non-traumatic cases, as subtle changes may reflect the severity and outcome of coma [15]. For instance, assessing brainstem reflexes can help detect severe conditions, such as uncal or downward cerebellar herniation locked-in syndrome and vegetative state [16]. Secondly, 65% (n=145) of respondents were in favor of utilizing a different scoring system than the GCS for assessing respiration. In the context of comatose patients, the assessment of respiratory patterns may reflect underlying etiologies, such as Cheyne-Stokes respiration, central neurogenic hyperventilation, apneusis, cluster breathing, or ataxic breathing [17]. Early recognition of these patterns could provide clues on the underlying neurological condition and aid in tailoring management plans for each patient. Lastly, assessing intubated patients revealed the most pronounced preference for an alternative to the GCS, with 85% (n=190) affirming their willingness to use a different scoring system. This strong consensus underscores a critical shortfall in the GCS-its inability to accurately evaluate consciousness in patients who cannot communicate verbally. In the FOUR score, hand gestures are used instead of verbal responses, which facilitates the assessment of afferent language processing even in patients with endotracheal intubation, aphonia, or vocal cord trauma. Additionally, the GCS may lead to misclassification in cases of language disturbances, such as motor, transcortical, or global aphasia, whereas the FOUR score allows dysphasic but alert patients to be accurately assessed based on their actual responsiveness [18]. Overall, these findings showcase healthcare workers' interest in the development and implementation of assessment tools that provide a more comprehensive evaluation of the neurological status, which potentially leads to improved patient care outcomes.

Finally, a growing body of research on the FOUR score supports its reliability, credibility, and predictive value [18]. To maximize the benefits of the FOUR score as an additional and/or alternative tool for the assessment of consciousness, it is imperative to enhance practitioners' familiarity and expertise through comprehensive education and training programs. These initiatives should be inclusive, targeting both junior and experienced healthcare professionals to broaden their competencies in utilizing this tool effectively. Such educational programs are crucial for ensuring that the implementation of the FOUR score is both accurate and consistent across various clinical settings. Furthermore, the development of clear, detailed guidelines and protocols is essential to support healthcare professionals in integrating the FOUR score into their practice seamlessly, hoping to potentially enhance the overall quality of neurological assessments.

Limitations

This study had some limitations that should be addressed. First, the data were collected through an online questionnaire, which may have an expected response bias. In addition, respondents were recruited from several hospitals and had different background specialties and institutional levels of care and might not have access to advanced critical care units, where the FOUR score is mostly used.

## Conclusions

This study provides insight into the perceptions of healthcare workers regarding the FOUR score. The FOUR score coma scale is not a well-established scoring method among front-line healthcare workers in Saudi Arabia, highlighting the need for an enhanced awareness of implementing this scale. There is a readiness to adopt alternative systems for assessing consciousness and interest in comparative studies of various coma scales, suggesting that educational initiatives could be well-received. While promoting the FOUR score, emphasis on its ability to assess the level of consciousness in intubated patients should be considered.
